# The Nation's Fund for Nurses

**Published:** 1917-05-05

**Authors:** 


### THE MATRONS' AND SISTERS' DEPARTMENT

### THE NATION'S FUND FOR NURSES.

### A Tribute from the British Empire to British Nurses.

The Nursing Profession is the only skilled profession that is at this present time without organisation. It is inarticulate, or perhaps it would be more correct to say that it speaks with such a multitude of voices that none carries either weight or conviction. The College of Nursing has now been founded to provide a Central Organisation—representative of the Nurses, elected by the Nurses, speaking in the name of the Nurses, and sufficiently powerful to compel attention to the Nurses' views.

A Royal College of Nursing.

The College is in being and is making its influence felt; its amalgamation with the Royal British Nurses' Association has been approved by both bodies, and the proposed supplemental Charter and Bye-Laws are now before the Privy Council. When this amalgamation is completed the Conjoint Bodies will be known as the "Royal College of British Nursing," and will already have nearly 10,000 Members on their joint register.

But no Institution of this kind can do without money, and, on the other hand, no Institution can get money without showing clearly the uses to which such money as may be obtained will be put.

An Endowment Fund.

The College is primarily intended to fill a place in regard to the Nursing profession similar to that filled by the Royal Colleges of Physicians and Surgeons in respect of the two Professions of Medicine and Surgery. In order that it may effectively take this position it is necessary to have an Endowment Fund. It is also desirable that inducement and facilities shall be given to those who show special aptitude for the higher branches of Nursing, and this necessitates the provision of Scholarships which will enable the recipients to pursue special courses of study or to travel with a view of familiarising themselves with the method of dealing with Nursing matters in other countries.

Nurses in Sickness and Old Age.

Besides caring for the education and training of the Nurses, the College also aims at helping the Nurses in sickness and old age. The Nursing Profession is a hard one, and many fall by the way. There are not many years in a Nurse's life when she is earning sufficient money to enable her to put by anything for illness or old age. It is hoped that the generosity of the public may enable the College to make provision for those of its members and others who, through no fault of their own, are in need or in suffering, thus repaying to the Nurses themselves some part of the vast debt owed to them by the community to which they have ministered so devotedly.

The College Buildings. Examination Rooms and Club.

Lastly, it is hoped that money may be forthcoming to provide a suitable building in London where the College may have its Headquarters, and where there may be Examination Rooms and Club Rooms where Nurses may meet.

The British Women's Hospital.

The College has, therefore, work in front of it which will need a large sum of money, and it has been decided to raise a Central Fund from which these various branches of work may be financed. This Fund is called "The Nation's Fund for Nurses," and the College is fortunate indeed in having secured the powerful support of the British Women's Hospital.

The Story of the Nightingale Fund.

In the "Life of Florence Nightingale" the story of the Nightingale Fund is told. One extract seems to have peculiar interest at this moment, when the continuation of Florence Nightingale's work is in contemplation, and especially when help is being so generously given by the British Women's Hospital:

Public meetings in support of the Nightingale Fund were held throughout England and the British Dominions. Among the speeches made at these meetings one of the most notable was Lord Stanley's at Manchester. "There is no part of England," he said, "no city or county, scarcely a considerable village, where some household has not been comforted, amidst its mourning for the loss of one who had fallen in the war, by the assurance that his last moments were watched, and his worst sufferings soothed, by that care, at once tender and skilful, which no man but only women could have shown. True heroism is not so plentiful that we can afford to let it pass unrecognised.

"Miss Nightingale has opened to her sex a new profession, a new sphere of usefulness. I do not suppose that in undertaking her mission she thought much of the effect which it might have on the social position of women, yet probably no one of those who made that question a special study has done half as much as she towards its settlement. A claim for more extended freedom of action based on proved public usefulness in the highest sense of the word, with the whole nation to look on and bear witness, is one which must be listened to and cannot be easily refused."

The Nation's Support for the "Nation's Fund for Nurses."

Surely the Appeal which met with such a generous response in 1856, after the Crimean War, may now well be renewed on a far wider basis. The British troops in the Crimea, in whose sufferings the foundation of nursing was laid, never numbered more than 70,000 men. To-day the British Armies at home and in every zone of the wide-flung battle-line are counted in millions; wherever the Armies are, there are the hospitals, and it is in the name of the Nurses in those hospitals that we ask for the nation's support to the "Nation's Fund for Nurses."

**Subscriptions and Donations may be sent to "The British Women's Hospital," 21 Old Bond Street, W. 1, or to The Secretary, "College of Nursing," 6 Vere Street, Cavendish Square, W. 1.**

